# The impact of curation errors in the PDBBind Database on machine learning predictions of protein–protein binding affinity

**DOI:** 10.1093/database/baaf061

**Published:** 2025-09-24

**Authors:** Jason D Gans, Justin E Miller, Esen Sokullu, Nileena Velappan, Ramesh K Jha

**Affiliations:** Bioscience Division, Los Alamos National Laboratory, Los Alamos, NM, 87545, USA; Bioscience Division, Los Alamos National Laboratory, Los Alamos, NM, 87545, USA; Bioscience Division, Los Alamos National Laboratory, Los Alamos, NM, 87545, USA; Bioscience Division, Los Alamos National Laboratory, Los Alamos, NM, 87545, USA; Bioscience Division, Los Alamos National Laboratory, Los Alamos, NM, 87545, USA

## Abstract

The PDBBind database has been widely utilized for the computational prediction of protein–protein binding affinities. While the accuracy of the PDBBind-curated equilibrium dissociation constants (*K*_D_) has been reported for the protein–ligand subset of the PDBBind database, the curation accuracy has not been reported for the protein–protein subset. Here, we present a detailed manual analysis for the subset of PDBBind records with PubMed Central Open Access primary publications and find that ~19% of these records had *K*_D_ values that were not supported by their primary publications. The impact of these putative curation errors on the machine learning-based prediction of *K*_D_ from experimental protein–protein 3D structures was evaluated and correcting the curation errors improved the Pearson correlation coefficient between measured and random forest-predicted log_10_(*K*_D_) values by ~8 percentage points. This finding underscores the importance of dataset accuracy for computational modelling and highlights the need for more stringent curation processes when extracting information from the scientific literature.

## Introduction

The accurate prediction of molecular binding affinity is a fundamental challenge in computational biology, with broad implications for drug discovery, protein engineering, and systems biology [1, 2]. Existing computational approaches for predicting binding affinity, including both machine learning and physics-based algorithms, rely on accurate experimental measurements of binding affinity to train and/or test models [3, 4]. The PDBBind database [5] is a commonly used dataset for this purpose, providing experimental data on protein–ligand, protein–peptide, protein–nucleic acid, and protein–protein interactions. Entries in the PDBBind link three-dimensional (3D) experimental molecular structures stored in the RCSB Protein Data Bank (PDB) [6] with experimental equilibrium dissociation constant (*K*_D_) measurements extracted from the published scientific literature. These pairs of 3D molecular structures and corresponding *K*_D_ values have been used to train computational models for predicting binding affinity from 3D structure and/or text string-based (e.g. amino acid sequence, "Simplified Molecular Input Line Entry System" [SMILES]) molecular representations [1, 2, 7].

Various sources of error and bias complicate the computational prediction of molecular binding affinities. Examples include (i) experimental errors in both 3D structure determination and *K*_D_ measurements [8], (ii) biased input data that are neither independent nor identically distributed due to the overrepresentation of frequently studied families of interacting molecules [9, 10], (iii) failing to account for differing experimental conditions (e.g. temperature, pH, and salt concentration) used in both 3D structure determination and *K*_D_ measurement [11], and (iv) the failure to explicitly account for molecular solvation and dynamics [12]. Attempts to mitigate some of these sources of error and bias have included the use of ‘high-quality’ experimental data (e.g. X-ray structures with <2.5 Å resolution) [13], and clustering-based cross-validation (to prevent the overly optimistic assessment of machine learning performance when very similar molecules appear in both the test and the training sets) [10, 14]. However, even with these mitigation procedures, reported assessments of machine learning-based predictions of protein–protein binding affinity, evaluated using different subsets of the PDBBind, range from 0.55 to 0.68 for the Pearson correlation between predicted and experimentally measured log_10_(*K*_D_) values [15–20]. The low Pearson correlation values achieved by these existing machine learning algorithms suggest that additional sources of error may be confounding predictions. We hypothesize that *binding affinity curation error*—the errors introduced when extracting *K*_D_ values from the scientific literature—contributes to poor machine learning performance.

The PDBBind curation error rate, defined as the fraction of records in the PDBBind that have been assigned *K*_D_ values that are *not* supported by a published paper, has been reported to be ~1% for the protein–ligand pairs available in the PDBBind (circa 2005) [21]. An assessment of the PDBBind curation error rate for protein–protein pairs has not been previously published.

In this study, we manually assessed the quality of *K*_D_ values for the subset of 262 protein–protein PDBBind (version 2020) records that are associated with Open Access publications available from PubMed Central [22]. Our analysis reveals a substantial error rate of ~19%. We further investigated the impact of these putative curation errors on a random forest (RF)-score [9] based, random forest [23] machine learning model for predicting protein–protein binding affinity. By correcting curation errors in the Open Access subset of the PDBBind protein–protein records, we observed an ~8 percentage point improvement in the Pearson correlation coefficient between predicted and measured log_10_(*K*_D_) values. These results illustrate the importance of dataset curation quality on the performance of computational models for predicting protein–protein binding affinity.

## Results

**Figure 1.**
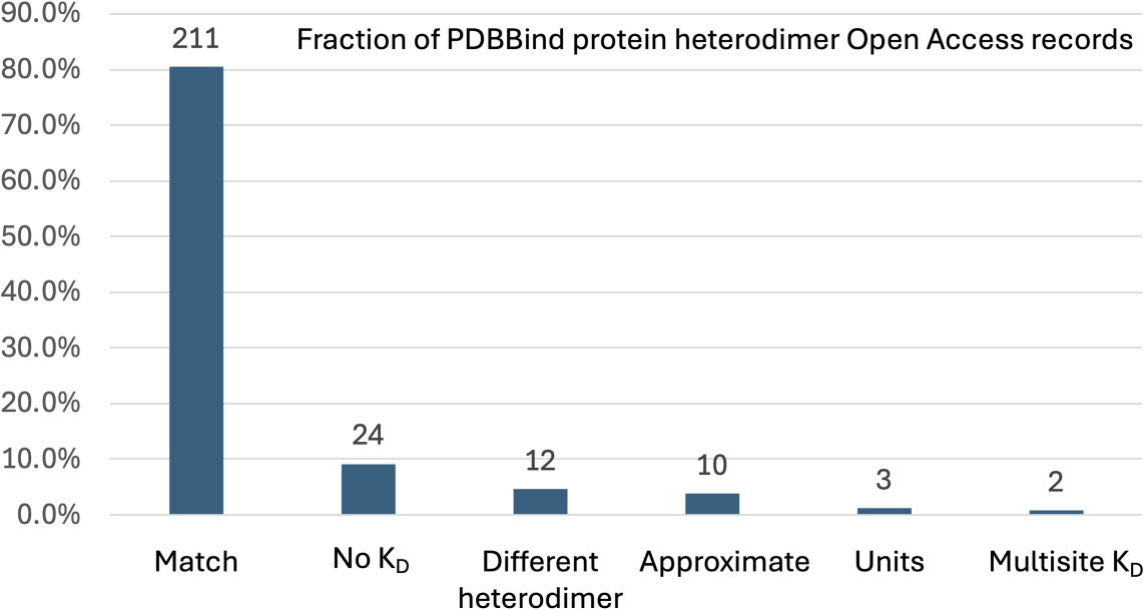
The fraction of PDBBind protein heterodimer Open Access records that belong to the curation categories listed in Table 1. The number of PDBBind records in each category is shown above each bar.

**Figure 2.**
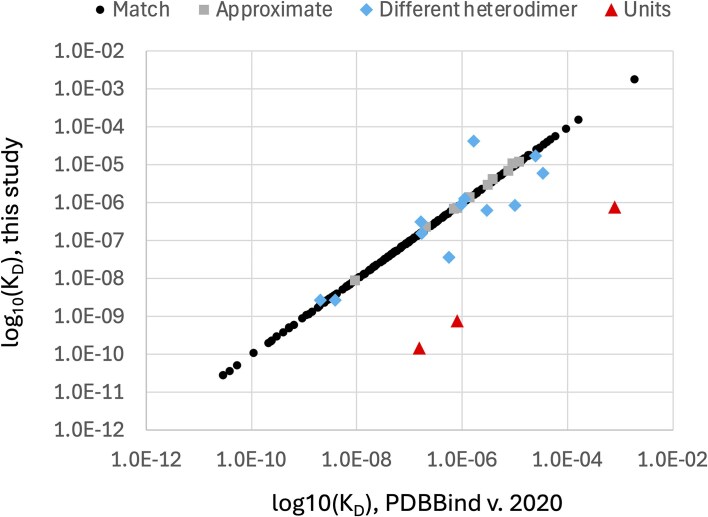
Direct comparison of PDBBind protein heterodimer log_10_(*K*_D_) values and log_10_(*K*_D_) values manually extracted from the literature by this study. Each point represents the *K_D_* values reported by the PDBBind and the *K*_D_ value reported by this study for a single protein heterodimer structure stored in the PDB with an Open Access publication. ‘No *K*_D_’ records are absent from this plot.

To evaluate the accuracy of the protein–protein PDBBind dataset (version 2020), we manually examined the subset of 262 PDBBind records that report protein heterodimer *K*_D_ values found in PubMed Central Open Access publications. Only the Open Access subset of PDBBind protein heterodimer entries were used by this study to enable bulk downloading from PubMed Central and to facilitate comparison with future, automated natural language processing methods for extracting *K*_D_ values. The *K*_D_ values manually extracted in this study (Supplementary Table 1) were compared to the *K*_D_ values reported in the corresponding records in the PDBBind. Our analysis revealed that 51 of the 262 PDBBind protein heterodimer records (19.47%) reported *K*_D_ values that do *not* belong to the associated protein heterodimer PDB record. This error rate is substantially higher than the previously reported 1% error rate for the protein–ligand PDBBind dataset [21]. To provide more detail on the nature of the curation errors, the *K*_D_ values reported by the PDBBind were manually assigned into one of six different categories, shown in Table 1. The fraction of the 262 protein–protein Open Access PDBBind records in each of these categories is shown in Fig. 1. A per-record comparison of the numeric *K*_D_ values from the PDBBind and those extracted by this study are presented in Fig. 2, excluding the ‘No *K*_D_’ category (for which this study was unable to identify a *K*_D_ value for the specified PDB record) and the ‘Multisite *K*_D_’ category (for which the pairs of *K*_D_ values reported by the publication were not directly comparable to the single *K*_D_ value reported by the PDBBind). As shown in Fig. 2, the ‘Units’ category contains only three records but has the largest individual *K*_D_ differences, which is not surprising since the commonly used units of molarity are ‘mM’ (millimolar), ‘μM’ (micromolar), and ‘nM’ (nanomolar), and incorrectly transcribing the units would cause an error of at least three orders of magnitude.

**Table 1.** Categories used to label the *K*_D_ values reported by protein–protein Open Access PDBBind records^a^

Category	Definition	Example
Match	The PDBBind *K*_D_ value exactly agrees with the value found by this study.	The PDBBind reports a *K*_D_ value of 2.49 × 10^−7^ M for PDB accession 4NL9 that matches the *K*_D_ value shown in fig. 4 of the primary PDB publication [24].
No *K*_D_	The protein heterodimer variant found in the PDB structure does *not* have a *K*_D_ value reported in the primary PDB citation.	The PDBBind reports a *K*_D_ value for PDB accession 6FC2. However, supplementary table S1 in the primary PDB publication [25] reports that *different* protein constructs were used for crystallization and ITC experiments.
Different heterodimer	The PDBBind *K*_D_ value belongs to a *different* protein heterodimer even though the primary PDB citation *does* report a *K*_D_ value for the associated PDB structure.	The PDBBind reports a *K*_D_ value of 1.1 × 10^−6^ M for PDB accession 4U0Q. This *K*_D_ value corresponds to the affinity of ‘full-length PfRH5 for basigin’. However, the PDB crystal structure is of ‘PfRH5ΔNL, encompassing residues 140–526 but lacking 248–296’ (see extended data table 1 in [26]) and has a reported *K*_D_ value of 1.3 × 10^−6^ M.
Approximate	The PDBBind *K*_D_ value is approximate and the primary PDB citation reports a more precise value.	Instead of reporting the two significant figure *K*_D_ value of 7.4 × 10^−7^ M for PDB accession 4CMM (from table 2 in [27]), the PDBBind reports the lower precision *K*_D_ value of 8 × 10^−7^ M (from fig. in [27]).
Units	The units of PDBBind *K*_D_ value do *not* agree with the *K*_D_ value reported by the PDB primary citation.	The PDBBind reports a *K*_D_ value of 1.5 × 10^−7^ M for PDB accession 5JSB. However, fig. 2 in the primary PDB publication [28] reports a *K*_D_ value of 1.5 × 10^−10^ M.
Multisite *K*_D_	The PDBBind provides a single *K*_D_ value, while the PDB primary citation reports two *K*_D_ values derived using a multisite binding model.	The PDBBind reports a single *K*_D_ value of 1.2 × 10^−8^ M for PDB accession 3GNI. The primary PDB publication [29] reports two *K*_D_ values, 2.5 × 10^−6^ M and 1.2 × 10^−8^ M (see fig. 6E in [29]) determined using a two-site binding model.

a The ‘PDB primary citation’ is the single published paper associated with each PDB record as reported by the Protein Data Bank.

Next, we assessed the impact of the different categories of curation errors on machine learning models trained to predict protein–protein binding affinities. Using a random forest regression algorithm with RF-score features [9] and a linear regression algorithm with Prodigy features [30], binding affinity prediction performance was quantified using the Pearson correlation between predicted and measured log_10_(*K*_D_) values using five-fold, clustering-based cross-validation [10, 14]. Since it is known that groups of molecules with similar structures and/or sequences can lead to artificial inflation of cross-validation-based assessments of model performance [7, 10, 14, 18, 31–34], clustering-based cross-validation was performed on groups of protein heterodimers, clustered using single-linkage clustering [35] and a Smith–Waterman pairwise amino acid sequence alignment-based [36] distance metric between protein heterodimers. Using a 3D structure-based distance metric between protein heterodimers (TM-align score [37]) did not significantly affect the observed Pearson correlation values calculated as a function of the number of clusters at each minimum separation threshold (data not shown). During clustering-based cross-validation, all protein heterodimers within a given cluster were collectively assigned either to the test set or to the training set. As the distance between clusters of protein heterodimers increases (controlled by the single-linkage clustering stopping criteria), the difficulty of the binding affinity prediction problem is expected to increase as the test and training sets become increasingly dissimilar.

**Figure 3.**
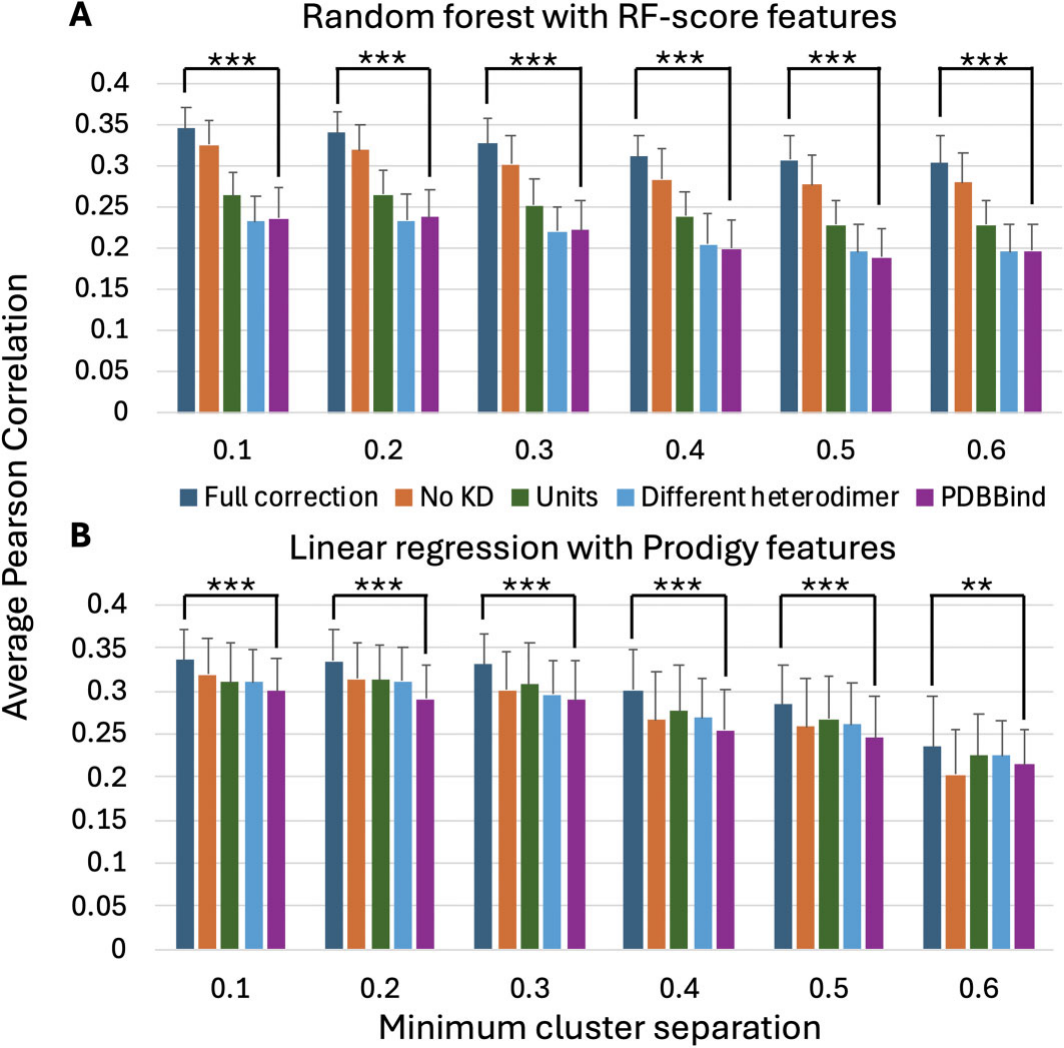
The average and standard deviation of the Pearson correlation between (A) random forest (RF-score features) and (B) linear regression (Prodigy features)-predicted log_10_(*K*_D_) values and log_10_(*K*_D_) values manually extracted from the literature for 100 independent trials of five-fold cluster cross-validation. Results are reported at six different minimum cluster separation thresholds for five different subsets of the PDBBind (v. 2020) Open Access protein heterodimer records. ‘Full correction’ (*K*_D_ values extracted from the literature by this study); ‘No *K*_D_ only’ (PDBBind excluding records assigned to the ‘No *K*_D_’ category); ‘Units only’ (PDBBind with corrections to the *K*_D_ values identified by this study as having units that do not agree with the primary citation); ‘Different heterodimer only’ (PDBBind with corrections to the *K*_D_ values identified by this study as having different values in the primary citation); and ‘PDBBind’ (PDBBind with unmodified *K*_D_ values).

To assess the impact of putative curation errors on machine learning predictions of binding affinity, random forest predictions with RF-score features [9] and linear regression predictions with Prodigy features [30] were computed for different categories of curation errors at multiple clustering-based cross-validation minimum cluster separation thresholds. As shown in Fig. 3, the Pearson correlation between predicted log_10_(*K*_D_) values and log_10_(*K*_D_) values extracted from the literature are higher for both the random forest and linear regression algorithms when using the *K*_D_ values extracted by this study than using the values extracted by the PDBBind (v. 2020). While the Pearson correlation values were small (<0.35 for both methods), the improvements in Pearson correlation of the ‘Full correction’ subset compared to the ‘PDBBind’ subset were statistically significant at all minimum cluster separation thresholds: Welch's *t*-test *P* < 10^−6^ for all predictions except for *P* < 10^−2^ for the linear regression at the 0.6 minimum cluster separation threshold. One-tailed Welch's *t*-test [38] *P*-values were computed for 100 independent trials of five-fold cross-validation.

Testing the effects of only applying corrections from individual curation categories selected from Table 1 shows that excluding the PDBBind records belonging to the ‘No *K*_D_’ category, followed by correcting the PDBBind records belonging to the ‘Units’ category, had the largest impacts on the random forest Pearson correlation. Since the clustering-based Pearson correlation values reported in Fig. 3 are computed for the Open Access subset of 262 heterodimer *K*_D_ values in the PDBBind, they are not directly comparable to the previously reported correlation values, e.g. 0.55–0.68, which were computed using larger numbers of PDBBind protein–protein heterodimer records with varying approaches for clustering-based bias removal [15–20].

## Discussion

The results of this study raise concerns about the curation accuracy of the protein–protein binding affinity values recorded in the PDBBind (v. 2020). Using the subset of 262 PDBBind entries that have associated Open Access publications, we estimate that the curation error rate in PDBBind protein–protein *K*_D_ values is ~19%, substantially higher than the previously reported value of 1% for protein–ligand *K*_D_ values [21]. The putative curation errors identified by this study are shown to negatively impact protein–protein binding affinity prediction performance of the random forest algorithm. These results highlight the need for more rigorous curation protocols and additional validation of datasets used to train and test machine learning algorithms.

However, it is important to note that only addressing curation errors is not sufficient to obtain highly accurate binding affinity predictions (e.g. Pearson correlation values between predicted and measured log_10_(*K*_D_) values ≥0.9). As shown in Fig. 3, the maximum average Pearson correlation value obtained for 262 Open Access PDBBind protein heterodimers at any level of bias-mitigating clustering is ≤0.35. Substantial improvement is still needed to enable reliable, highly accurate prediction of protein–protein binding affinity.

There are other potential sources of binding affinity prediction error that should be systematically evaluated by the scientific community. These include the effects of neglecting differences in the experimental conditions used to measure both 3D heterodimer structures and heterodimer binding affinities. In particular, it is common for the experimental pH, temperature, and salt concentration to differ between X-ray crystallization conditions and binding affinity measurement conditions for the same protein heterodimer from a single published study, and between different protein heterodimers from different published studies [3, 11, 33, 39]. Despite these experimental condition differences, many studies (including this study) only use a single protein protonation state (typically at pH ~7) for structure-based prediction of binding affinity [40–43]. As experimental conditions (other than the experimental 3D structure determination method) are not currently included in the PDBBind database, additional effort will be needed to collect and curate this information from the scientific literature. Finally, most machine learning-based affinity prediction methods do not include explicit solvation effects and do not include structural waters (which are available in many X-ray crystal structures).

## Materials and methods

### Dataset selection

The subset of 262 protein heterodimer complexes from the PDBBind (version 2020) database that are associated with PubMed Central Open Access primary citations were identified using the following multistep process. First, protein heterodimer records in the PDBBind were identified by computing the set intersection of heterodimer PDB accessions (obtained using an ‘Advanced Search’ of RCSB PDB with the query builder search: ‘Structure Attributes’ → ‘Assembly Features’ → ‘Oligomeric State’ + ‘is’ + ‘Hetero 2-mer’) and the PDB accessions from the protein–protein portion of the PDBBind (from PDBBind supplied file ‘INDEX_general_PP.2020’, which excludes peptide–protein complexes). Then, the resulting set of protein heterodimer PDB accessions were used as queries via the RCSB PDB REST API to extract PubMed identifiers for the primary literature associated with each accession. Finally, the PubMed identifiers were used to query the list of Open Access publications (from https://ftp.ncbi.nlm.nih.gov/pub/pmc/oa_file_list.csv) and download the full text and supplementary material associated with each Open Access publication using the PubMed Central OA Web Service API [44]. This study focused solely on PubMed Central Open Access publication to (i) allow bulk downloading of full-text publications in compliance with existing publisher restrictions, (ii) enable the *K*_D_ values extracted by this study to serve as a benchmark for future automated natural language processing approaches, and (iii) reduce the number of publications requiring time-consuming human curation.

### Manual curation

We manually reviewed each Open Access publication associated with a PDBBind protein heterodimer entry to identify an equilibrium dissociation constant (*K*_D_) corresponding to the associated PDB structure. The extracted *K*_D_ values were manually classified into the curation categories listed in Table 1 and are provided in Supplementary Table 1. As previously noted [13, 21], manual curation from the scientific literature is a challenging and time-consuming process. In particular, individual structural biology publications frequently present information for multiple protein–heterodimer complexes derived from (i) homologous proteins from different organisms, (ii) mutagenesis experiments to test biological hypotheses, (iii) the creation of novel molecular constructs to facilitate structure determination (e.g. truncation/deletion mutants, modification of glycosylation and acetylation, and mutations to add or remove disulfide bonds), and (iv) a range of experimental conditions (e.g. pH, temperature, and salinity). Identifying *K*_D_ values for a given PDB record frequently required the manual cross-referencing of amino acid sequence and per-residue modifications (e.g. glycosylation and acetylation) from the PDB record with the sequence information provided in both the publication main text and supplementary material. PDB records for which the protein construct sequences used to experimentally measure the *K*_D_ (as reported in the primary literature) did not match (due to amino acid substitutions, modifications, insertions, or deletions) the protein construct sequences reported in the RCSB PDB database were assigned to the ‘No *K*_D_’ category.

### Machine learning

A random forest regression algorithm [23] with RF-score features [9] was used to predict protein–protein binding affinities and assess the impact of putative curation errors in the PDBBind. RF-score feature vectors contained the number of atom pairs in 3D protein heterodimers structures for which the atoms belonged to different proteins and were separated by no more than 12 Angstroms. All hydrogen atoms and atoms with missing atomic coordinates in PDB files were ignored. Atom pair counts were binned by unique atomic element pairs for all the elements found in the input protein structures. All atoms belonging to protein amino acids and covalently attached chemical groups were included in the RF-score feature vectors. Water molecules and other chemicals not covalently attached to a protein were excluded. Each random forest model used 100 decision trees, and individual trees were constructed for a randomly selected subset of 50% of training PDB records by maximizing the variance reduction at each branch. The performance of each random forest model was evaluated by calculating the Pearson correlation between predicted log_10_(*K*_D_) values and log_10_(*K*_D_) values manually extracted from the literature (by the PDBBind or by this study) for 100 independent trials of five-fold clustering-based cross-validation.

The linear regression-based Prodigy algorithm [30] was also used to predict protein–protein binding affinities. The Prodigy feature vectors consisted of:

- Counts of the number of pairwise ‘charged–charged’, ‘charged–polar’, ‘charged–apolar’, ‘polar–polar’, ‘polar–apolar’, and ‘apolar–apolar’ inter-residue contacts between the different heterodimer protein chains.
- ‘% Non-interacting surface’ (%NIS) for polar, apolar, and charged residues.

All Prodigy features were computed using the Prodigy python code (https://github.com/haddocking/prodigy). Training set feature selection was performed for each cross-validation fold using the Akaike information criterion (AIC) selection process (forward and backward) as described in Vangone and Bonvin [30]. The feature selection methods in Vangone and Bonvin [30] were modified to include choice of distance threshold (ranging from 3.5 to 20 Å, in increments of 0.5 Å) for counting inter-residue contacts in the AIC selection process. This modification ensures that selection of the distance threshold is not influenced by the test data.

The following subsets of protein heterodimer complexes from the PDBBind with Open Access publications were used to train and test a random forest model:

- ‘Full correction’ (236 heterodimers): Excluding the 24 heterodimers in the ‘No *K*_D_’ category and the two heterodimers in the ‘Multisite *K*_D_’ category from the 262 heterodimers curated by this study.
- ‘No *K*_D_ only’ (238 heterodimers): Excluding the 24 heterodimers assigned to the ‘No *K*_D_’ category from the initial 262 PDBBind-curated heterodimers.
- ‘Units only’ (262 heterodimers): Starting from the 262 PDBBind-curated heterodimers and correcting the three associated *K*_D_ values identified by this study as having *K*_D_ units that do not agree with the primary citation.
- ‘Different heterodimer only’ (262 heterodimers): Starting from the 262 PDBBind-curated heterodimers and correcting the 12 associated *K*_D_ values identified by this study as having different *K*_D_ values in the primary citation.
- ‘PDBBind’ (262 heterodimers): The unmodified PDBBind-curated heterodimers with associated *K*_D_ values.

### Clustering-based cross-validation

Clustering-based cross-validation has been used in predictions of protein–ligand binding affinity [10, 14] to prevent an overly optimistic assessment of machine learning performance when very similar molecules appear in both the test and the training sets. As with protein–ligand binding affinity prediction, publicly available data for protein–protein binding affinity prediction are also biased by the inclusion of many highly similar protein heterodimers (i.e. homologous proteins from different organisms, mutant protein sequences created to test specific biological hypotheses, etc.). The presence of similar protein heterodimers (with similar *K*_D_ values) in both the test and training sets can inflate the machine learning performance metrics estimated using traditional cross-validation on individual protein heterodimers. To help mitigate this bias, clustering-based cross-validation groups all available protein heterodimers into clusters and assigns individual protein heterodimers within the same cluster to either the test set or the training set, but not both. Clustering was performed using the single-linkage clustering algorithm [35] with the following sequence alignment-based distance metric between pairs of protein heterodimers, {*p_a_, p_b_*} and {*p_α_, p_β_*}:


(1) \begin{eqnarray*}
&& {\mathrm{Distance}}\left( {\left\{ {{{p}_a},{{p}_b}} \right\},\left\{ {{{p}_\alpha },{{p}_\beta }} \right\}} \right)\\
&& = \frac{1}{2}{\mathrm{min}}\left( {d\left( {{{p}_a},{{p}_\alpha }} \right) + d\left( {{{p}_b},{{p}_\beta }} \right),d\left( {{{p}_a},{{p}_\beta }} \right) + d\left( {{{p}_b},{{p}_\alpha }} \right)} \right)\\
\end{eqnarray*}

(2) \begin{eqnarray*}
d\left( {{{p}_i},{{p}_j}} \right) = 1 - \frac{{{\mathrm{SW}}\left( {{{p}_i},{{p}_j}} \right)}}{{\sqrt {{\mathrm{SW}}\left( {{{p}_i},{{p}_i}} \right){\mathrm{SW}}\left( {{{p}_j},{{p}_j}} \right)} }},
\end{eqnarray*}

where SW(*p_i_, p_j_*) is the Smith–Waterman pairwise sequence alignment score computed using the BLOSUM62 scoring matrix [45]. The clustering distance metric presented in Equation (1) was adapted from the protein–ligand distance metric presented in [10] and is independent of both the order of protein heterodimers (i.e. ${\mathrm{Distance}}( {\{ {{{p}_a},{{p}_b}} \},\{ {{{p}_\alpha },{{p}_\beta }} \}} ) = {\mathrm{Distance}}( {\{ {{{p}_\alpha },{{p}_\beta }} \},\{ {{{p}_a},{{p}_b}} \}} )$) and the order of the individual proteins within each heterodimer (e.g. ${\mathrm{Distance}}( {\{ {{{p}_a},{{p}_b}} \},\{ {{{p}_\alpha },{{p}_\beta }} \}} ) = {\mathrm{Distance}}( {\{ {{{p}_b},{{p}_a}} \},\{ {{{p}_\alpha },{{p}_\beta }} \}} )$). The minimum cluster separation was recorded after each iteration of single-linkage clustering.

## Supplementary Material

baaf061_Supplemental_File

## Data Availability

All software for this effort is available from https://github.com/LANL/BIORAD under the BSD-3 open-source license.
